# Publishing publicly available interview data: an empirical example of the experience of publishing interview data

**DOI:** 10.3389/fsoc.2024.1157514

**Published:** 2024-06-05

**Authors:** Diana Enriquez

**Affiliations:** Department of Sociology, Princeton University, Princeton, NJ, United States

**Keywords:** open source, qualitative methods, interview data, secondary data, archival data

## Abstract

In September 2021 I made a collection of interview transcripts available for public use under a CreativeCommons license through the Princeton DataSpace. The interviews include 39 conversations I had with gig workers at AmazonFlex, Uber, and Lyft in 2019 as part of a study on automation efforts within these organizations. I made this decision because (1) I was required to contribute to a publicly available data set as a requirement of my funding and (2) I saw it as an opportunity to engage in the collaborative qualitative science experiments emerging in Science and Technology studies. This article documents my thought process and step-by-step design decisions for designing a study, gathering data, masking it, and publishing it in a public archive. Importantly, once I decided to publish these data, I determined that each choice about how the study would be designed and implemented had to be assessed for risk to the interviewee in a very deliberate way. It is not meant to be comprehensive and cover every possible condition a researcher may face while producing qualitative data. I aimed to be transparent both in my interview data and the process it took to gather and publish these data. I use this article to illustrate my thought process as I made each design decision for this study in hopes that it could be useful to a future researcher considering their own data publishing process.

## 1 Introduction

A few weeks before I published my interview data online, I announced through Twitter that I had a set of interview transcripts with gig workers from Amazon, Uber, and Lyft that I intended to publish for public use. I did this as a first step in my distribution effort. I know distribution is a numbers game: the more people who know the data exists, the more likely it is to reach the handful of people who need it. What I had not anticipated, however, was how excited Academic Twitter became about this news: within 24 h, my tweet passed 200,000 views and the thread was filled with requests from other academics and UX researchers who asked me to share the link to the data set once I posted it. I learned that within academic and industry settings, there is a lot of interest in public qualitative data sets and many questions about how one would make recent qualitative data public. While many people were excited and grateful to see my announcement, some researchers have concerns about the privacy of the subjects and the value other researchers can derive from interview transcripts read outside the original researcher’s fieldwork (Daries et al., 2014).

These data were the product of a larger study on pre-automation and experiences with self-driving vehicles. I began this project with a team of five researchers. I handled the design and gathered the interview data for this study with input from the PI and my other colleagues. My interviews were a piece of a larger project that relied in part on publicly accessible survey data, patent data, and my qualitative fieldwork. My co-authors on the larger project included three quantitatively focused researchers in addition to my qualitative work, which was overseen by another qualitatively focused PI. This larger project produced three articles using different parts of the project’s complete data gathering effort.

One condition of our funding for the initial project was that we contribute some of our original data to a public archive. Increasing numbers of funders are encouraging this kind of output from studies with the hope that the data can aid other researchers in their work. As a group we determined that the interview data was the clearest data asset for us to contribute to an archive. Within our overall data collection efforts, the interviews were a new data contribution in a rapidly changing field, whereas the quantitative data was already publicly available information. Once we began our study design, I needed to consider what it meant to create a meaningful asset to the publicly available qualitative data. Every design decision I made about the fieldwork – from the first draft of the interview guides through publication – was made with an eye toward risk assessment for the interviewee and the desire to produce a meaningful public asset.

There are many debates about open science, including debates focused on qualitative research. Some debates focus on the practice of open science and the value of collaboration production of new ideas (Hughes, 1993; Cowan, 1999; Becker, 2008; Aad et al., 2015). Some debates focus on improving transparency and reproducibility in sciences overall (Travis, 1981; King, 2011; Elman and Kapiszewski, 2014; Moravcsik, 2014; Aguinis and Solarino, 2019; Shaw et al., 2019; Pratt et al., 2020; Jacobs et al., 2021; Nosek et al., 2021). Others focus on the ethics of publishing qualitative data (Wax, 1977; Parry and Mauthner, 2004; Mauthner and Parry, 2009; Mauthner and Parry, 2013). Others theorize about the potential value secondary data has for open science (Heiskala, 1998; Wallerstein, 1998; Corti, 2000; Moore, 2007; Abu-Lughod, 2008; Heaton, 2008; Knorr-Cetina, 2013; Tsai et al., 2016; Freese and Peterson, 2017; DuBois et al., 2018; Feldman and Shaw, 2019; Ruggiano and Perry, 2019; Class et al., 2021). Though these are important debates, they are not the focus of my article. I reference some of these debates as I elaborate on my thought-processes and my design decisions, but I am more focused on what decisions I made as I prepared to publish my interview data. When I decided to publish my data, I had trouble finding a step-by-step account of how to publish it and what design decisions I needed to make along the way. The purpose of this article is to provide that step-by-step example I needed in case it is useful to someone else considering making their own qualitative data contributions.

My position is that the qualitative researchers who study technology and other rapidly changing fields will need to work more collaboratively to work effectively. One way to improve collaboration may be to share qualitative and quantitative data more frequently with both other academics, as well as the journalists and policy makers who are expected to record these historical moments and design appropriate policies. The data we gather is also useful to the researchers inside companies like the ones I consider in my study: while internal UX researchers may not be able to demand funding or working time to study the issues workers face in automated workplaces, my data provides a historical record of their experiences. The record itself may be useful for designers and engineers inside firms who are thinking about the best ways to improve worker experiences alongside the more finically focused goals of their product managers.

Through careful design and on-going conversations with respondents, I believe there are responsible ways to gather and publish some kinds of qualitative data. I agree that not all qualitative data can or should be shared publicly for a variety of reasons that I will discuss. I want to be clear: I designed my study with the intent of publishing the interview data but not the more informally structured qualitative data I gathered to support and corroborate my interview findings. I argue a crucial piece of secondary data analysis is completing some form of additional data collection to compare and corroborate qualitative data sets as a method of analysis in new studies. I present my case as an example of how someone could prepare their data for a public archive, as well as design choices I would make differently next time.

In this article, I explain:

1. my thought process at each stage of the design and implementation of my study,
2. how I prepared these data for publication,
3. how I published and distributed these data,
4. and the use cases that emerged once these data were public.

I conclude the article with the lessons I learned as I implemented my project and what I might do differently next time. I hope this step-by-step discussion is useful to others who are considering their own data publishing processes.

## 2 Why I was motivated to share my data

I decided to publish my interview data for two reasons: (1) I was required to make a produce a public data asset as a requirement from our funder and (2) I believe in the efforts emerging in Science and Technology studies to produce more collaborative, real time research.

Even before the funder’s requirement, I was motivated to publish my interview data because I am committed to the work made possible through collaboration and comparison of qualitative data through emerging “open science” experiments. Collaboration between researchers already happens within Science and Technology studies as well as Anthropology (Ducheneaut, 2005; Shamir, 2010; Heller et al., 2011; Collins et al., 2016; Collins, 2017). These modern cases of collaborative qualitative research, especially around rapidly changing fields like Science and Technology, demonstrate that there is value in interpretive collaboration within qualitative social science. It may, in fact, be necessary. As an important example: in Vertesi’s (2015), a team of scientists collectively interpret images of Mars to develop a system of building knowledge for an environment they will likely never experience outside the data collected by their rovers and the images processed by their computers. As Vertesi describes: these images, as a type of qualitative data, require this kind of collective narrative building because the data itself is so far from our own contexts that it requires a team effort to interpret. At its core, this is a scientific example where scientists must work with limited data to inform their future experiments and data gathering processes. Another example of a collaborative method in ethnography was the “swarm ethnography” method developed by Goodman and Vertesi (2012). Their method was designed to capture the full picture of a technology in a social context through simultaneous, fast-paced qualitative work with the intent to contextualize several different researchers’ data and analyses alongside one another. While each researcher managed their own interviews and gathered fieldnotes, they collectively had to make decisions about how to format and present their fieldnotes to the whole team alongside their analysis. Their effort to package their fieldnotes and analysis in consistent formats would improve their ability to compare data and analysis across each interviewer’s contributions. These are two examples of collaborative qualitative work that benefit from collaboration between researchers and their data sets.

My data and research are also grounded in the methods and literature of Science and Technology studies as much as they are grounded in Sociology. I chose to study how workers reacted to technology in their workplace, knowing the specific kind of worker I wanted to study was often very pressed for time and difficult to access. Given how quickly the technology evolves and how difficult these studies can be to conduct, I decided early on to share my data as a potentially useful snapshot of a technology and human reactions to the technology in time. To me, publishing my interview data as my data snapshot alongside the articles where I introduced my analysis of the field was a useful contribution to a rapidly changing field. I want to invite other researchers to collaborate with me and compare our interpretations over time.

## 3 Part one: designing a study with the intent to publish interview data

There were many design decisions I needed to make upfront once I decided I was going to publish my interview data. These decisions felt important because I know that not every data set should be made publicly available. I was determined to figure out what a public data set that was respectful of my interviewees’ privacy and still useful to a broader audience could look like. The questions I considered in my design choices are not meant to be exhaustive – they should be seen as a case that moved from design through implementation and into the aftermath of publishing public data.

## 4 Design decisions I made during my interview design

I strongly believed that I needed to make careful design decisions about my fieldwork and the final interview dataset from the beginning. This felt like a crucial step toward defining and managing risk for the interviewee. The safest way to gather the data I needed and assess risk for interviewees seemed to be through semi-structured interviews. The risk assessments would be translated into my consent forms and discussed with interviewees before we began. I explain here how I approached the design of my interviews and assessed my hypothetical data to determine which pieces were publishable and which seemed too risky.

I chose to focus on publishing my interview data, rather than the fieldnotes I assumed I would collect, because I believed a structured interview set would be easier to (1) manage for potential risks to interviewees and (2) work with as an artifact in a data archive. When I define my interviews as “structured data,” I mean I wrote a list of specific questions I wanted to ask. While there could be some variation in how the final question was worded, which would allow me to use the same kind of technical terms my interviewee preferred to use when describing their jobs and the technology they used at work, the intent and meaning of the question were clearly defined. The interviews would be short and efficient (15–20 min with three sections: one about their work experiences, one about their technology at their gig work job, and the final section focused on their perceptions on the future of technology). In contrast, I describe a “semi-structured interview” as an interview with structured questions and additional, flexible time in the interview for more thematically defined questions that would adapt to the specific person. I did not leave time for this kind of undefined interview portion – my intent was to ask each interviewee the same questions.

The clearer structure of our conversation thus gave me a clearer sense of what risks could arise with each question. Through structured interviews, the cases are a little easier to compare and the structure helps me corroborate company/organization-related events or technology features someone describes to see if what is described to me is a common occurrence in that moment of time or an unusual case. Given the triangulation I already do with my interviews, I hope someone else later could work with the structured data in their own triangulation efforts and/or trace specific themes over time (DuBois et al., 2018). Thinking about these future potential use cases among scholars impacted the way I thought about the structure of my data. I did not design my fieldnotes with the intention of publishing them as a public asset, which I will explain later in this article in the section on preparing data to be published.

## 5 Establishing a consent protocol

The depth of qualitative data and the relationships we build in our field sites give us access to intimate details of people’s lives. Within these relationships, some data are more sensitive than others. As researchers, we can anticipate some aspects of an interview that could be sensitive topics for an interviewee. Other sensitive topics may be difficult to predict. I tried to think carefully about how to communicate and manage risk for my interviewees.

### 5.1 Assessing risk for interviewees

One risk I know researchers face as we conduct research with living respondents is that legal constraints can limit participant privacy in unexpected ways. A case where an interviewee described planned violence against another individual, for example, would mean I was legally obligated to report information to law enforcement (Weiss, 1994). Another potential risk was that my publicly available qualitative data could include information that causes trouble for the individual if their testimony became part of a criminal case. While we as researchers can sometimes anticipate these issues and file for exemptions, we cannot always predict what someone will share with us nor whether it might become interesting to law enforcement (Khan, 2019). In my assessments of risks, I determined that I did not anticipate these issues as serious risks in my brief and structured interviews because I was focusing on (1) the interviewee’s legal work experiences, (2) their interactions with existing and legal technologies in their workplaces, and (3) their perceptions of hypothetical technologies like self-driving cars.

A more difficult step in my risk assessments was about how to describe privacy risks that come with published interview transcripts that could be accessible to anyone on the internet. Privacy on the internet is an increasingly challenging obstacle for researchers and private citizens. Murphy et al. note the pressure ethnographers face to switch from traditional handwritten notes to more shareable audio files and other digital formats of fieldnotes (2021). Moving toward sharable digital files presents new concerns about managing participants’ privacy (Murphy et al., 2021). No matter how many data security measures researchers take, we know from the frequent data leaks at large companies that there are limits to privacy when participant information is stored digitally (Balebako et al., 2013; Ragab et al., 2021; Leonardi and Neeley, 2022). Even survey data could be used to trace information in the shared data sets back to respondents (Narayanan and Shmatikov, 2009). Digital ethnographers face even more extreme privacy challenges: because much of their fieldwork occurs in a searchable public domain, it is particularly difficult to maintain participants’ anonymity (Geiger and Ribes, 2011; Shklovski and Vertesi, 2013; Reich, 2015; Enriquez and Vertesi, 2021).

I believe concerns about privacy and other risks associated with sharing recent data exist on a spectrum. There are different kinds of issues and degrees of severity of risk that exist with fieldnotes, interviews, and survey data. There is also greater risk to individuals sharing personal data (i.e., financial, or medical data) vs. more observational data (i.e., how does this kind of technology work, how do you do your job). Knowing I would be sharing our interview data when this project was completed, I developed a discussion guide that was focused on the interviewee’s experiences at work and with the technology they were using. With the design of the guide in mind, I could explain the kinds of questions I wanted to ask them as I established a consent protocol. It is easier to consent to something more concrete (including specific kinds of questions I wanted to ask) than it is to ask for consent to observe someone and then publish my notes about their behaviors, for example.

### 5.2 Communicating risk to interviewees

One consideration I had as I designed the study is that most adults are now used to hearing or reading interviews in public media – whether this is on the radio, in a news story, or through a family member’s school assignments. Collections of interviews come in many different formats for public consumption. For example, collections of interviews, like Nobel Prize winner Svetlana Alexievich (2017), present a curated series of interviews that together paint a picture for the reader of what it was like to experience the collapse of the USSR. Public projects like the Story Corps allow individuals to upload their own oral histories, interviews, and conversations into a public forum for anyone to use.^1^ Oral histories are frequently sampled from library collections and public forums like Story Corps to produce radio shows like the BBC’s “Listening Project”^2^ and NPR’s Story Corps channel.^3^ I introduce all these very public cases to demonstrate that the process of gathering and listening to oral histories and interviews is a very common experience, and our interview subjects are very likely to be familiar with the format. I could mention these examples of publicly available interviews to interviewees while explaining where the interview transcripts could appear in an archive to a student or someone else hoping to read the interviews. When one is familiar with the format and the final product, it is easier to consent to than when the final product is as distant to them as an academic article, for example.

There are debates in qualitative research about whether an interviewee could fully assess the risks associated with publishing their full interview transcripts. Many ethical debates focus on the harms that could occur and that respondents may not want their data to be public, but they often omit the idea that maybe the respondent would like their full transcript available because there are also harms in being misrepresented or presented out of context (Tamminen et al., 2021). I am uncomfortable with the assumption that our adult respondents cannot comprehend the risk of sharing their experiences or opinions enough to consent to have their interview transcript published in full in a public archive. The full transcript does not necessarily feel riskier to me than using some of the deeply specific segments of interviews as quotes in academic articles, which carry their own risks for being similarly traceable. In practice, even in sensitive contexts where the subjects are asked about their health, many respondents are willing to contribute their data to other research projects beyond the current project because it is perceived as a public good (Yardley et al., 2014). Some researchers argue that publishing data, as a product co-produced by the researcher and the respondent, should not be the choice of the researcher alone (Parry and Mauthner, 2004). I agree – consent to publish data is necessary. However, given their role as co-producers, adult respondents should have agency to decide whether their structured interview data becomes public. We may even consider whether interviewees should have a right to demand that their full interview is made publicly available alongside the selected quotes an author presents with their article.

While I could explain some privacy risks upfront, it is necessary to remind interviewees that we cannot predict every possible use case of a public interview transcript. Several scholars argue that it is impossible to state or predict every possible outcome of research or archival data – after all, if we knew every outcome, there would be no need to conduct the research (Bishop, 2009). Discussing the outcomes as some cases we can predict and others we cannot is honest. It may also be necessary to remind the respondent that once something is on the internet, it is nearly impossible to control how someone interacts with it. I see these as features of modern conversations about informed consent in any protocol because they apply to the brief quotes and descriptions selected for papers as much as they apply to a full interview transcript.

### 5.3 Designing the consent protocol

I decided that consent was never going to be a single conversation with the subject. Consent and privacy are a multi-party process, rather than a single moment (Cutliffe and Ramcharan, 2002). There are also active moments of gathering consent, engaging with the interviewee’s comfort level throughout a conversation, and providing the interviewee time to reflect after a conversation and before interview transcripts are published. The direct and indirect moments where someone could choose to revoke consent are important – consent in the abstract may be challenging to give, but consent after an interview is completed and an interviewee has time to reflect can be useful too.

My conversations about consent often happen in several stages during an interview. The first occurs during recruitment, when an interviewer presents information about a study and the potential interviewee decides whether to pursue more information. The second occurs when more information about the study is presented to the potential interviewee and an interview is scheduled. Third comes with a formal conversation about consent – ideally as both a consent form that is reviewed and signed by the interviewee AND a verbal consent review before the interview begins. Fourth is the opportunity for an on-going consent reminder/check in if there are uncomfortable moments during an interview. And finally, there is a consent remind at the end where the interviewee is offered time to reflect on their responses and the interview experience. Interviewees are presented with contact information to request redactions in the interview text or file complaints before analysis and data are published.

The first set of design decisions about the interview structure and, subsequently, what I was asking a subject to consent to happened in a loop. I considered the kinds of questions I wanted to ask, assessed how likely the question would be answered with deeply individualized and traceable responses, and then considered how I would ask someone for their consent to share that information with me and the broader public. This interview guide development-consent conversation editing was iteratively produced as I prepared my application for the IRB. My final questions were structured to pose minimal risk to interviewees. This project allowed itself to be more anonymous because I was looking for common work experiences and interactions with technology.

As I anticipated risks to the subjects, I knew that there could be peripheral details in each case that might be sensitive to the individual to discuss. I focused my questions on their experiences at work and interacting with a piece of technology assuming these questions would not be traumatizing or especially sensitive to the subject, given how often these two topics are normalized in discussions one might have with a relative stranger as a form of small talk in social settings. Asking about work could be sensitive in cases where some of the work is illegal or stigmatized, but I was specifically interested in legal forms of gig work completed by publicly traded companies – this means the company and the work completed have some degree of oversight by federal regulators. As I recruited subjects for the interviews, I asked them screening questions about their jobs and told them what I intended to ask about (1) their gig work experiences and (2) their experiences with technology. This screening process helps interviewees manage their expectations at this first consent conversation, where a potential interviewee decides whether to request more information about the interview opportunity.

During my recruitment process, I described the study as a project about gig work experiences and experiences with technologies at work. I was clear that I was not trying to gather personal information about the individual, nor would they be compared to other respondents based on sensitive characteristics like race or religion. While there are many cases where extreme care toward privacy and confidentiality is important, I would argue there are other cases where the descriptions of an experience are so commonplace that the text itself would be extremely difficult to trace back to an individual. In my case, I needed to find a lot of respondents who had common experiences with the technologies they were using so I could describe the technology. I could explain upfront to the workers that I was trying to learn about a specific kind of work and a specific kind of technology through their experiences and perceptions. The biggest concern I anticipated was a worry that a less worker-friendly firm like Amazon might discover an AmazonFlex driver had spoken to me about their experiences. When I brought it up later with some workers during our conversation about consent, several of them noted there are so many workers in so many warehouses with so much turnover that it would be hard to track it to any one person.^4^

The formal consent process consisted of a document I sent to interviewees before the interview for review and their signatures. This consent form reached them through the email address they used to contact me as we scheduled their interviews. I verbally reviewed this consent form at the beginning of our interview and again at the end of the conversation as a less formal check-in to see how the interviewee felt about the conversation. During the consent description at the top of the interview, I also gave them examples of how I would scrub their interviews to remove identifiers and trails back to them. I mentioned I would check in with them informally throughout the conversation if they seemed uncomfortable, and I reminded them that we could skip questions or remove answers if the interviewee expressed any hesitation to a question. In this way, I tried to remind interviewees that consent was both an active conversation and one where I, as the interviewer guiding the conversation, needed to practice reading the room. Consent is not always easy to give, which meant I also needed to read the non-verbal cues of discomfort.

At the end of our conversation, I reminded them that they had resources like my PI’s contact information and the IRB office information for Princeton if they needed to register a complaint. I also reminded them that they had my email address if they left the interview and decided they wanted to strike anything from the conversation from the final record. I decided that interviewees should be reminded that there is a lag between interviewing and publication – which gives them time to reflect on an interview and assess any negative experiences or risks they associate with the now completed experience. Sometimes, the period of reflection helps someone weigh the risks a little more easily than they could in the abstract. This final form of consent is more passive and open ended – I presented it as an opportunity to contact me, my PI, or the IRB if they changed their mind or had any questions. If they did not write to us, we are led to believe their earlier moments of direct consent remain valid. Maybe there should be another point of checking in with them when the interview is published, but I did not offer this final step for this particular project. By the end of our conversation, the interviewees and I had several points of contact about consent.

### 5.4 Reviewing the consent protocol with the IRB

As part of my consent design process, I had several conversations with Princeton’s IRB. I knew that before I gathered any data, I had to explain to Princeton’s IRB what kind of data I wanted to collect and why I wanted to make the data public. This required me to explain how the risks to the respondents compared to the public good of making our data publicly accessible. My direct and more hypothetical conversations with the IRB officers helped me navigate the interview guide-consent guide iteration process I mentioned as I designed my consent protocol.

Rather than seeing this as a challenge to be “negotiated” with a hostile regulatory group, I treated this as an opportunity to discuss the possibilities of what I could do in the field and with our data after the project. As an example of what I explained to the IRB: in evaluating the risk of gathering and later sharing this data, my goals were to be (1) minimally invasive in their work days, (2) specific in the kinds of work procedure and technology questions I was asking them, (3) clear that the workers did not need to answer any questions they did not want to answer, and (4) clear that the workers were free to add whatever additional context they felt was important to share with me as they answered my questions.

I argued there could be risks in describing the future of automation in their workplaces, given the existing precarity of their work and the stressful ideas of futures where steady work was even less accessible, but we hoped the interviews we were gathering could help us demonstrate more clearly the role that these workers play in making technology work correctly. While Uber, Amazon, and Lyft may advertise themselves as technology companies, at least the delivery side of their work relies very heavily on the intervention and improvisation of the human workers completing tasks (Shestakofsky, 2017; Enriquez and Vertesi, 2021; Vertesi et al., 2021). The public benefit to making these interviews accessible was (1) academic and (2) allowed the workers themselves to provide their own narrative of their work experiences to a broader audience.

## 6 Part three: gathering data

### 6.1 My interview data

The qualitative interview data I published included 39 transcripts of interviews with gig workers from AmazonFlex, Uber, UberEats, and Lyft. I published these data with a ReadMe that provided context on the study that led to this round of fieldwork.^5^ As a basic summary overview, the interviews consisted of a near-even split of 21 female drivers and 19 male drivers. One transcript was omitted from the final interview pool because I discovered he was lying about his gig work experience. Because I ran recruitment ads through FreshEBT and Facebook, I reached gig workers across the United States, including 25 workers in urban settings and 15 rural workers. While I produced ads and discussion guides in English, Spanish, and French for this study, all the interviews I completed were in English at the preference of the interviewee. I include these details to demonstrate what kind of meta data we decided to include with each interview because these items were visible to me but often not discussed directly in the interview transcript.

I made the decision to drop an interview from the published data set because the interviewee was lying about being a gig worker. I am not the first qualitative researcher to navigate an interview where the interviewee is clearly making something up, either with the intention of telling me what they thought I wanted to hear so they could collect the promised compensation or because it was entertaining to them to do so. Owens (2022), for example, describes encountering a “professional research subject” during fieldwork and how the respondent’s responses were different enough from the others in the study that it was clear the respondent joined the study not as a relevant source, but as someone hoping to give the interviewer whatever they wanted to hear. In my interview data set, this subject was clearly lying because the claims he made about his work life varied wildly from sentence to sentence and did not correspond to the descriptions of any of my other research participants. By the end of the 20 min, it was clear this person had not interacted with the gig worker version of the apps I was studying. Owens’ case of a “professional research subject” was recruited from a community with a similar economic status as my own catfishing research participant.^6^ Since this case was not an accurate representation of the data I intended to collect, I dropped his case from the final published data set.

Within this process of evaluating the validity of the interview, I realized it could be difficult for someone less familiar with the field site and unable to hear the hesitations and mid-word contradictions of the interviewee over the phone. The reader would be able to see my attempts to untangle some of his conflicting statements and provide clarity, as I experimented with my hypothesis that he was lying about his work experiences but left room for him to clarify his points and show me he had specific examples of how he worked with the technology despite my growing doubts. In the specific clarifying questions I asked, it is clear this interview is different from the others. I still decided to drop the interview from the final data set because I did not think it provided useful insights on the topic I intended to cover with the data when I published. Upon reflection, given the use case that emerged where professors were using the transcripts in classrooms to teach students to code data, it could also have been a useful tool in how to evaluate the validity of qualitative data. I will note that in the critiques of interviews, there are many concerns about people lying or exaggerating in their responses. From this case I present and Owen’s example, there are ways to validate interview data that are very difficult to do with standardized, lower social friction data like surveys.

### 6.2 On-going engagement with consent

As I anticipated, I found in my consent-focused conversations with interviewees, it makes it easier for subjects to consent to their data moving into the public domain when they are familiar with the format and some of its use cases.

Each interviewee and prospective interviewee were emailed a copy of the consent form before our interview began. The consent form is included in the Appendix. I also read and talked through the specifics of the consent form at the beginning of the call (especially around the privacy protocols and that we would publish the transcript when the project was completed). I talked to them about how I think about and clean data. This was to ensure the interviewees understood my intended uses for the data in my study and that they understood the tools I offered them if they were uncomfortable and wanted to withdraw from the study.

One consideration moving forward: I described the audience for the interviews as “researchers” in the written consent form. To academics, the assumption may be that “researchers” indicates other academics. When I verbally reviewed the consent form with each interviewee, I explained to them that these data were going in an academic archive, but anyone could see them. I listed some of the people who would use them but also said there may be other people outside of universities who read them, like journalists. I found that my interviewees interpreted “researchers” to mean anyone conducting “research,” which could include students and journalists, without it becoming a stressful distinction. For future versions of the consent form, it may be better to write out that possible audience members include “generally curious individuals on the internet” to establish that the audience would not just be academics. Some were excited about this possibility; the rest gave neutral responses (for example: “ok,” “sounds good,” “go ahead.”) encouraging me to continue to the interview portion of our conversation. Many of the interviewees returned signed copies of the consent form before we met, though some sent the letters after we met, and I reminded them to submit the letters. All interview subjects consented or reconsented at the beginning of the call verbally before I began the interview. I checked in throughout the conversation, especially when someone provided some personal backstory, to make sure they were still comfortable.

Our consent forms explicitly stated our intention to make the interviews available for public use and offered the interviewees the chance to request that specific parts of their answers be omitted from the final public copy. We often frame data sharing as solely a risk for participants, but I learned in my consent conversations that many of our participants were glad we planned to share their interview transcripts with students and researchers. They were excited that their stories were going to be used in broader research efforts. One interviewee told me about several times throughout our conversation that he was proud to participate and share his story. It clearly meant something to him that his specific story, in his words, was going beyond my research files and into a space for students. This desire to help students and provide evidence from personal experiences is something I frequently encounter with my interviewees across my studies. The interviewees explained that participating and contributing to data that helps researchers and students learn about the world felt like a benefit, as we hoped it would be perceived to be when we filed our IRB application stating our intentions to make my data public.

In some ways, I expected this conversation about publishing the transcript of our full conversation alongside my academic article to be a positive one. I argue that in the age of social media, where more people are more conscious of their public presence, there are cases where our data is not especially sensitive and we are better equipped to consider what it means to have information about ourselves online (Marwick, 2015). I find subjects sometimes get nervous about being “quoted out of context.” Thus, offering to make the full conversation available along with my article and my selected quotes from their responses might reassure some respondents who feel better when their full story in their own words is available for corroboration. As a subject later told me, they felt comfortable knowing their stories would be there along with the “rest of [their] own words.”

Further, when I checked in with my subjects at the end of the conversation to ensure they were still comfortable sharing their responses, many of them stated they were eager to be part of a public conversation about their working conditions. Several of them mentioned they were more interested in participating because their data “could help students.” My project was not especially interesting to them, but they liked that the interview could help a broader community of researchers. The results of my conversations about adding the co-produced data to archives are not unusual: even cases of completed studies where an archive contacted respondents directly to ask for their permission to add their interview transcripts to an academic archive were met positively by most respondents (Cutliffe and Ramcharan, 2002; Bishop, 2009).

This especially enthusiastic participant, along with several others, chose to disclose other personal information about themselves as additional context outside the scope of the initial discussion guide, but the interview guide and general project goals were not designed with the intention to gather sensitive personal data. When some of our conversations grew more sensitive, I listened and gently tried to guide them back toward the specific questions I mentioned we were trying to discuss. I used the time constraints I promised them (that the interview would be about 20 min) as one way to remind them that I wanted to respect their time and the scope of the interview, but they were welcome to take the time they wanted to add context. I hoped this added reinforcement was helpful for reminding them about the nature of the study and would give them a minute to reflect on whether they wanted me to know the information they were sharing. In the end, two of the interviews, in particular, concerned drug use/recovery. One of the subjects chose to move away from the topic after he made a specific contextual point he felt was relevant to my questions and the other took the time to reflect on why he was completing the kind of work he was doing now.

### 6.3 Other omitted data: the choice to omit my fieldnotes

I gathered several different kinds of fieldnotes at different points in the study and omitted all of them from the final published data package. While I took great care to consider the structure and goals of my interviews with an eye toward publishing the interview transcripts, it seemed impossible to structure and assess risk for my fieldnotes in quite the same way. I use fieldnotes as a flexible method to (1) help me review my fieldsite and interviewee recruitment strategies, (2) help me corroborate information presented to me about technologies and/or the structure of a job, and (3) to keep track of news related to these topics shared through social media channels and discussion boards. I typically prepare my fieldnotes into more coherent summaries of information gathered over time for presentation in an academic article. The information I gathered is never completely listed in an academic article, but the analysis is. Further, my fieldnotes for this project were usually not related to specific interviewees themselves, and thus, it did not make sense to attach them to a specific transcript as an addendum with the text. Further researchers may consider what form fieldnotes should take to be shared more publicly and what risk it carries to present them alongside a set of interview transcripts.

In addition to the interviews, I also completed a digital ethnography component to support my interview data for my articles. I spent 1 year gathering fieldnotes observing discussion threads in three Facebook groups that serve as crowdsourcing communities designed and operated by Amazon Flex workers, many of whom also drive for Uber or Lyft. As a general method strategy: I often try to include an ethnographic component in my projects to either help me understand the language and processes I want to ask about directly as I structure my interviews OR I complete the ethnographic work after my initial, focused interviews to corroborate my initial findings. The hypothesis behind the latter as a method choice, given that I study strategies and uses of technology, is that the issues/strategies my interviewees tell me they use are likely visible in the digital community spaces they participate in through the digitally recorded entries of other community members’ behaviors (referred to as “digital traces” by Geiger and Ribes, 2011). In the way that ethnographers might do a first round of fieldwork, begin writing, and then return to the field for a more targeted round of research, I used my digital ethnographic work as a very focused look at the types of technical complaints, their frequency, and the responses that Flex drivers introduced to help each other address their problems (Small and Calarco, 2022). This complimented my interview findings and allowed me to extend my data. I include this description here as a case of how one may decide to supplement my interview data with their own fieldwork extension.

The fieldnotes I gathered during the interviews served as the first step in my analysis – I focused on connecting points between different interviews, noting differences in interpretation between interviews, and notes on language (how specifically someone described a technological feature). I did not share these notes because they were a draft of the analysis, which was later refined into an article and shared publicly in that prepared format. I had other fieldnotes from the beginning of the study that I gathered as I was figuring out how to enter the field site. As noted in the description of my data and fieldsite, it is very challenging to get this kind of worker on the phone. Gig work means that the connection between time and money is especially pronounced. We also live in an era with increased phishing and scams, so I had to be careful about how and where I contacted people offering a small amount of compensation for a few minutes of their time for an interview. As I made multiple attempts to contact people through different settings, I made notes on what was or was not working and why I suspected it worked or did not work. These specific methods notes again made it into my article as a summary of methodological choices, so I did not share the fieldnotes.

The final type of fieldnotes I gathered were around the patterns of conversations that occurred within public Facebook groups run by AmazonFlex drivers. These notes were the least structured kind of fieldnotes I gathered and often it was focused on revisiting questions/points made by my respondents in interviews. As one way to check how frequently an error or issue may occur, I sometimes reviewed the discussions around specific technology or working conditions in these groups. My hypothesis was that if an error/experience a respondent described in an interview was common, it should not be difficult for me to find similar discussions within these central community spaces. My fieldnotes from these secondary review processes were frequently lists of tallies of how often something came up in conversations, variations between different perspectives in the group and my interviews, and cases where the point a respondent made seemed to be a very isolated case. It helped me determine which points in my interviews I would share as the core evidence in my article. As a discipline, our position on how/when we consent and use public digital discussions is still under debate. While I had alerted everyone in these groups to my presence and reason for being there more than once, it is hard to consent everyone on every conversation within these massive groups. My primary purpose for gathering this kind of data was also to structure my analysis and corroborate my findings, so the interviews were still the clearest commentary I could provide to respondents.

I could control and anticipate the design of my structured interviews much more closely than I could anticipate my fieldnotes and ethnographic findings. This made it much easier to structure a conversation about risk and consent when I asked my interviewees for permission to publish our interview transcripts. I was already nervous about the potential response from researchers when I published my structured interview data, so taking an additional leap to try to structure and publish unstructured, adaptive fieldnotes in their rawest form felt too risky to attempt this time and would be an interesting experiment in a future study.

## 7 Part four: preparing the data

### 7.1 Reflecting on the audience

Given how quickly technology changes – and so do perceptions about what a technology does or does not do – I knew my interview data would become a historical, rather than present, record on some aspects of the information I was gathering. Capturing data quickly and frequently is especially important in Science and Technology studies, where the regulatory environments and technologies of interest can become practically unrecognizable in a matter of years or even months (Leonardi and Barley, 2008; Wajcman, 2015). Thus, it became useful to think about the final output as a submission to an archive first and a current sociological data set second. I saw this because I think the next project where my interviews are useful is first in a comparison of changes over time using snapshots like mine and second, as a tool for designing future sociological studies or developing hypotheses for future projects. I suspected the data would be useful to journalists studying gig work in in-depth reporting contexts or for the many academics I knew who transitioned from socio-technical work into industry settings and were trying to improve worker-facing interfaces for these emerging technologies. While the exact data may be outdated for these latter two cases, it could help them develop hypotheses for future research questions in similar ways to sociologists and historians. Thus, I was guided by some of the principles developed by historians and other qualitative researchers who focus on archival work and thought about the kind of information I could preserve in the present that would make my snapshot more useful to them later (Fielding, 2004).

With all this in mind, it was useful to me to think about the historians and other archival researchers who might be interested in my data to answer different kinds of questions. The Oral History Association has provided historians with guidelines and protocols on how to preserve and share data since 1968. Historians who record interviews frequently do so with the intent to add their data into historical archives after they publish their work (Ritchie, 2014a). Sharing interview data in library archives, in their view, contributes to the permanent record of a historical moment and thus offers (?) opportunities for others to interact with the data to produce new theories or alternative interpretations (Bishop, 2005, 2007, 2009, 2014; Ritchie, 2014b). These principles were useful to me in the design stage because I could see how researchers worked with an object once it was further away from the initial moment where the data were produced. I could see how historians and other archival researchers triangulate between different artifacts to make sense of a historical moment, and I could try to anticipate some of these needs in the structure of my interviews and final meta data produced.

### 7.2 Cleaning data and masking

Designing the data-anonymizing protocol is part of the IRB review and the consent review process. Other challenges emerged once I needed to complete the data masking process. Some edits (like proper nouns) are easier than others. One especially important issue I considered: this data is co-created between me and the interviewee. When I choose to omit something, am I giving the interviewee space to represent themselves as they wanted to represent their perspective?

With qualitative data, each element removed comes at a cost. The more context or details about the subject that are removed, the more difficult the data would be for another researcher outside the original field site to analyze the final data set (Jerolmack and Murphy, 2019). Thus, I weighed each element of an interview transcript by (1) risk to the individual, (2) value to the context of the document, and (3) the structure of the data set overall, specifically whether there are opportunities to provide useful context about the data set as a whole that is less compromising to the individuals in the data set than direct quotes, while still providing useful field information to a new researcher.

In the larger project, we had two coders work with the data for our articles, so it seemed appropriate to have more than one person read through and mask the data as an added security precaution. Our masking efforts focused on removing direct and indirect identifiers (Kapiszewski and Karcher, 2021). When we first produced the transcripts, we had the transcribers sign NDAs as they listened to the audio files. We asked two undergraduate research assistants under NDAs to review and remove personal identifiers from the transcripts before I returned to the transcripts and removed any lingering identifiers. Each undergrad research assistant on our team did the first read and removed data they considered sensitive or identifying.

This first pass generally removed the direct identifiers – proper names and sometimes names of towns/cities. I went back through each transcript for a second read and removed other indirect identifiers like landmarks or indicators of specific locations within a state (though geographic regions, like rural Connecticut, were allowed to remain) (Kirilova and Karcher, 2017). I also removed regionally specific businesses that could be used to identify location when the businesses were especially concentrated in a geographic point on a map, and any other unique case-related identifiers. For example: in cases where the individual held a series of jobs that included nationwide companies AND a local small business – I would abstract the small business by describing it by its function rather than its name. As another example, some respondents described going to a regional grocery store chain and I removed the name. I also removed information like how frequently the regional grocery store chain was available in their specific location, replacing it with an abstracted description of the business such as (common midwestern grocery store chain) to provide useful information without indicating an exact location. Overall, my strategy for masking my data for archival use reflects many of the recommendations made by Corti et al. (2000) given their experience with the UK’s Qualidata archive.

After this standardized approach to cleaning and anonymizing the data, I returned to these two transcripts that presented issues because they contained discussions of more sensitive topics than I expected to gather given the scope of this project. I mentioned my concerns to my co-author: that these transcripts included references to drug use/recovery that we could argue were relevant to the study, but we could also argue were outside the scope of the study. In one case, the interviewee references drug activity to establish himself as an expert in identifying some patterns of behavior among passengers in his car. In the final interview transcript, we discovered that the audio quality was poor at that moment in time in the interview, so the words are hard to understand in the audio file unless you were part of the initial conversation. We did not need to omit them in the final script because the most specific details are caught as [unintelligible]. In the second transcript, the interviewee describes longer term addiction and his process toward recovery. The work he was doing at the time was a step in his recovery, which is why he wanted to share his background with me. We decided to keep the stories about his drug use, homelessness, and the limited resources he was able to access as an army veteran in the transcript because he was very clear throughout the interview that he was proud to participate in the study and he hoped his story could help others who found themselves struggling. My digital ethnographic work also revealed that these two cases of Uber drivers were unlikely to be easily identifiable based on their veteran status or past drug use because there are many drivers who were veterans and/or individuals recovering from substance dependencies. When we considered the sensitive data to be part of the context around when/why someone accepted this kind of gig work role, we could provide a better description of the drivers and their circumstances, which was a goal of this qualitative research. For these reasons, we kept the long descriptions about mental health, substance use and recovery, PTSD, and other conditions that the driver describes.

From these cases, my co-author and I had an interesting conversation about what it meant to participate in the study and how much of the consent process could be defined by us in advance vs. what the interviewee wanted to contribute to answering our questions. Was there a point where I needed to reconsent the interviewee if I wanted to include all the data he wanted to share with the study, even if it felt out of the scope of our interview goals? I had already decided to drop the interview case of the interviewee who was lying about his experience as a gig worker. Should I omit sections of this interview because they were out of scope? Was this a step in what it meant to “clean data” for public consumption, in the ways a survey data researcher might omit noise from a data set? We decided to keep this section of the interview in the original transcript because a clear argument could be made that these detailed stories about his past life were part of his decision to drive with Uber at the time. They were also details that, while specific and possibly identifiable traits, are unfortunately common stories about the workers who come to rely on gig work platforms for employment when many of their financial and other living conditions are in flux (Auguste et al., 2022). We determined that the interviewee had repeatedly said he wanted students to know his story, and when I reminded him I could remove information from the final transcript, he said again that this story was important to him to share. While I had a goal for the kind of data I wanted to collect, I felt a responsibility to honor his request as the co-producer of his interview data.

## 8 Part five: publishing the data

We used existing infrastructure through our university to host our data, rather than develop our own infrastructure as a team or a department. Princeton created “an online repository designed for archiving and publicly disseminating digital objects which are the result of research, academic outputs, or administrative work performed by members of the Princeton University community.”^7^ There is a lot of debate about the kind of screening tools that should exist between a potential user and the data set as a form of security: many researchers recommend that potential users should register with the archive and declare how they intend to use the data (Bishop, 2005; DuBois et al., 2018). This may be controversial, but I will explain my choice: our platform does not require users to register themselves to access my interview data.

While there are many emerging data archive platforms, I chose this platform because I was familiar with the support my interview data would receive through my institution’s library. Princeton’s library system promotes open access and would continue to maintain the platform after I left the institution. This library, like many other academic libraries, has dedicated librarians who manage the data and help students find the resources they need for projects. Librarians are important partners in maintaining and distributing the data in our growing digital archives (Mannheimer et al., 2019). This institution, compared to a government-managed platform, was small enough for me to know who the data stewards were and to give them my input on how I wanted the data to be hosted and distributed. In this sense, the platform is a community effort within my university setting. The DataSpace team is also committed to making the data easy for students and other researchers to discover, which was important to me. There were mutual benefits for me as a researcher publishing in an environment that provided me with a DOI link and for the university library to have a new data set that drew in a lot of public traffic. As some researchers mention, the broader public responds favorably to researchers providing data for public use in an accessible way (DuBois et al., 2018).

Given the small scale and local setting of our archive platform, I had frequent conversations with the DataSpace curators to ask for advice as I prepared my data. New items are reviewed and the packaging around these items is edited by a set of DataSpace curators as a team within the Princeton Research Data Service. These curators pay particular attention to the information included in the ReadMe files that accompany each item as one way the digital objects are introduced to new audiences. Beyond the packaging, these curators also reserve a DOI and provide guidelines on how to cite the materials in other contexts.

The ReadMe file became the introduction to the data set and our attempt at “Meta data” for the interview collection. We worked with the repository’s curators to determine what kind of data was necessary to include in the ReadMe. This was largely informed by the other data sets on the website and the standards determined by the organization within Princeton that set up the repository. The repository is formatted such that each transcript is its own PDF that someone could download as an individual file. The ReadMe file offers general information about the “author” (me), a summary of the data including how many files there are and when it was gathered, how to cite the data, information on articles written using this data, and a codebook for how each file is labeled. I included details about the field site methods, including how the subjects were recruited and what criteria were used to select them in our screening process. Our interview guide was simple, given how brief each interview needed to be, so I included the list of structured questions at the bottom of this document rather than attaching a new file as “interview guide.” Finally, in this file, I explained the consent process and the anonymization process for each file before we published the data. Our intended audience was not a very specific category as we prepared the Meta data text. I assumed primarily students and/or departments teaching interview coding strategies might be the first to use the data. As this was my and my co-author’s first experience with open qualitative data, we were curious to see where the data would go and had few specific expectations.

I realize my decision to have it in a public, non-restricted forum may seem controversial. Many researchers argue that the respondent’s privacy is best protected by data enclaves and other platforms that require registry for use (Bishop, 2005; Field et al., 2021). First, it is the policy of the platform I chose to leave the door fully open and not require users to register themselves before they access data. I accepted this policy because there are already so many barriers between academia and others outside academia that I wanted to choose an environment that welcomes more casual exploration of my data without the users needing organizational credentials to access the transcripts. Sometimes the organizational credentials themselves feel too intimidating for someone to register, even if this line of identification is not required. I wanted to make sure people like my respondents could find and read their transcripts without feeling self-conscious about the space, even a digital one, they entered. I embrace the idea that analysis and discussion of the topics I study should happen outside purely academic research environments. Beyond my own considerations, other researchers note that qualitative research is often meant to generate, rather than test, theories (Haven and Van Grootel, 2019; Field et al., 2021). Not every researcher who approaches my data will have a clear study that they intend to conduct using my data. My data was published during the COVID-19 shutdown, thus, some of the heightened interest in my data was probably due to the heightened demand for data that could be accessed remotely (Corti, 2000). Overall, I would like to normalize more exploratory research, especially when recent qualitative data sets like mine are accessible through archives.

It was important to me to find a platform where the data would be free for broader public use, thus, I was interested in pursuing a platform that used a CreativeCommons license for free usage of my data so long as the user cited the original source of my data (Mannheimer et al., 2019). I selected the CreativeCommons license because it was the format I was most familiar with – it is a system designed to encourage sharing and reuse. The fact that my data would be accessible to anyone, regardless of their education-affiliation or background, was appealing to my respondents, given their own varied backgrounds of education and socio-economic class. I worried more restrictive licenses that prevented redistribution, for example, would violate my promise to my respondents that their data would be broadly accessible. Beyond the requirement that the user cite the original data so others can find the data, I did not pick something more restrictive that would divert resources away from the platform to enforce it. The CreativeCommons license allows them to see and use the data for free while making it easier for others to find their way back to my data archive.

## 9 Part six: emerging use cases of the published data

Before I published the data, I assumed the interviews would be useful to instructors in classrooms teaching their students about how to code data. Though the students were not in the field with me as I produced these data, they can practice “decontextualizing” the data and finding other ways to interpret my data against the other sources of information they have (Moore, 2006). My assumption was further supported by Bishop and Kuula-Luumi’s (2017) study of how qualitative data sets from the UK Qualidata repository are typically used; teaching and research were more common uses among graduate students and professors, while undergrads primarily downloaded the data set as an opportunity to apply what they were learning in class to a final project.

Another use case I expected was from other researchers who practice triangulation in their fieldwork: Qualitative researchers approach their work with different goals in mind based on their understanding of what their research offers. Those who are excited about my data may be supportive of the idea of contributing to general knowledge through collaborative and simultaneous projects. Others who conduct qualitative exploratory research before they attempt larger scale quantitative studies also expressed interest in my data and the process of publishing these data, even when my topic was outside the scope of their research. The final group interested in my decision and process to publish these interviews seemed to be those who follow their quantitative work with qualitative research (DuBois et al., 2018).

Others who are more skeptical of what my data could offer may be more focused on understanding a particular phenomenon in its context. Researchers who are more skeptical of the value of interview data may reject the value of these published interviews, arguing that they lack the deeper context that participant observation or ethnography provide (DuBois et al., 2018). Some researchers express concern that methods of analysis like grounded theory do not allow for the same structured hypothesis testing and review at the core of natural sciences (Glaser and Strauss, 1967; Corti, 2000). Again, I stress that the design of the overall data from the beginning matters greatly and that treating my data set as a case study in a moment of time/design of a specific technology makes it useful in some cases.

Now that the data have been publicly available for a year, the use cases I am aware of include: (1) use in universities’ classrooms to teach students about interview coding and analysis, (2) undergrads completing independent research projects using these data for their analysis, (3) public media stories about the conditions gig workers experience at work, and (4) community organizations using these data to corroborate issues they have identified within their own work experiences. In the first two cases, the data provided a more hands on experience for undergrads to engage with qualitative data. The second two cases were more specific to the year when I released my data and may be less relevant over time (especially in the case of embargoed data). Another case is likely to emerge in the future when historians return to historical data about gig work in the time before the drastic changes that occurred during Covid-19 and the battles over gig work classification.

The most active use case was within methods classrooms. As instructors added the data to their methods courses, they tweeted sections of the interview transcripts and credited me to describe their classroom discussions from earlier that day. Several of them referred to my data sharing and maintenance as “service to the field,” which may be one way to anticipate the additional stewardship that became necessary with the public data set. One example of this unexpected stewardship occurred when the hosting website was down for the day and several instructors sent me direct messages to my Twitter account to ask if I had intentionally hidden the data or if there was a technical issue with the website.

At this point, one of my articles using these data was published and the other was under review with a journal (Enriquez and Vertesi, 2021; Vertesi et al., 2021). I acknowledged the risk it could pose to me if I published my data before my second article was published. I decided, however, to respect the wishes of our funder rather than continue to embargo my data until an unclear final date. The instructor asked if I would share both of my articles using the data so she could walk her class through interview coding and theory building using my articles as the end case. At this point, I had to decide how to manage a public data set against the timeline of my other articles. I concluded it might be easier to publish public data AFTER I had finished using it for my own articles. In this case, we released it before my second article was published to maintain our agreement with our funder and because we did not feel that our article would be threatened by the release of our data.

I had the bonus experience of watching how my data was interpreted by UX researchers in technology companies – from my own time within these organizations, I know there was a lot of hesitation around reusing data. I would argue training in how to reuse and triangulate data is currently very limited, thus adding to the overall suspicion around using existing data for current research questions. Some researchers are tackling the problem head on and expanding on the methodological literature around secondary data analysis. Kern and Mustasilta (2023) define secondary research analysis as using primary data generated for a different project to answer new substantive questions. Several researchers offer clear processes through real data on how one can work with secondary qualitative data effectively for a study: Bishop (2007) recommends the researcher begins by “understanding context, defining a subject area, finding data and sampling, later sampling and topic refinement, and relating to transcripts.” Bishop notes that an important part of contextualizing archival data is understanding who the researcher was and what that meant for the interaction between respondent and interviewer – and describes how this tricky relationship can be discussed in the analysis section of an article. This analytical statement is not a challenge reserved for only researchers conducting secondary analysis: Fielding (2004) argues that the interviewer and the researcher using the archival data are both tasked with describing the role and influence of the interviewer in the co-production of data. Chatfield (2020) recommends approaching the data flexibility and knowing some degree of mixed data or method may be necessary to answer the substantive question guiding the researcher through the data. Kern and Mustasilta (2023) describe how to reconcile different interview data sets as “cases” for comparison. Together, these articles provide enough for a methods lesson on using secondary analysis (?) that is valuable in a wide variety of contexts.

Throughout the year, I did my best to provide the additional context and resources as use cases for my public data set emerged – including many disclosures about myself as an interviewer. I saw this as part of my role as a steward of the data. The students and professors who wrote to me with questions about the data helped us think through the other kinds of meta data we might provide with our transcripts. These series of interactions painted a picture for me of what to expect in the maintenance of my data set but also in the ways my presence as something embedded in the data would continue to require my interaction with the researchers who chose to engage with my data set. A handful of college seniors emailed me directly to thank me for the data and sent along abstracts or outlines on how they might want to use the data for a project. Their follow up questions reflected researchers trying to add in the fieldwork context they knew was important as they read the interview transcripts I shared with them.

For example, one college senior wrote to me asking about the gender and general location of some specific speakers. She was studying gender and ideas about entrepreneurship, so she wanted to understand the social context of the speaker. Some of the undergraduates remained in touch for the entire year while they worked on their projects and provided regular updates on their analysis and progress. Very politely, one of them asked me if I would be willing to talk to her so she could have a little more context on my fieldwork and the tone of some of the interviews she read already. Another student asked me if I would share additional demographic information about the different speakers because, as the transcripts were written, it was hard to tell the race, gender, and general location of these individuals. After discussions with my co-author, I decided to share a table with each interview listed by gender and state. I omitted race, because while I had this data available on some of the interview subjects, there were others I could only guess from other context in our interview or from the profile pictures they used on whatever electronic payment tool we used to transfer the compensation I promised them for participating in the study. The student who requested the gender and location data began a project focused on some of the political leanings expressed in the interviews, a topic very distant from the original intentions of my data collection and the articles I produced from these data. She believed these traits could be important to help her form her conclusions. Her questions to me in the following year introduced one of the clearest cases of someone using the data to answer a very different kind of question than the data was originally gathered to answer. With the support of her academic advisers, she found a way to work with my data to answer a very different kind of research question.

## 10 Part seven: conclusion: lessons and future considerations

I still believe that publishing my interview transcripts required both (1) upfront planning and (2) post-publishing adjustments as use cases for the data emerge. Ideally, by sharing my planning process and the cases I encountered after I published my data, other social scientists will have more information available to them as they plan their own public data releases. In summary, before I gathered data, I developed a clear sense of what kind of data I wanted and weighed the risks to the subjects of gathering this data with the intention of making it public. While I did not consult the DataSpace curators in the overall design of my project, it may be practical for other researchers to discuss the study design with an archive’s managers early on. Particularly, the librarians and others involved with maintaining an archive and distributing data may have useful insights on what works/does not work with an open data collection (Mannheimer et al., 2019).

During the fieldwork, I focused on clear consent protocols and did my best to keep the scope of the interviews within the guidelines of the discussion guide, knowing the discussion guide was where I had developed my risk assessments. These ethical debates around informed consent are important, and I agree that not all data should be added to a public archive – especially without a conversation about consent with the respondent. I disagree, however, with claims that informed consent is impossible and that subjects cannot comprehend what it means to have their interview published. While I was initially worried it may turn respondents away, it turned out to be a useful tool for recruiting respondents.

My experiences in gathering consent and publishing the interview transcripts lead me to believe it may be necessary to ask more respondents what they would like to have happen with their data beyond my academic outputs. I think there are interesting questions to consider about whether researchers should need to make their data public if the respondent requests it, given the respondent helped create the data. Does the respondent have a right to request the publication of their full interview? Afterall, the benefit of an academic article to the respondent is often minimal, but it may be seen as a public service or good if their interview can then be used in classrooms or to inform government policy. For some respondents, it may also be useful to have the full interview context available if they feel they were misrepresented by a quote taken out of context in an academic article.

Once the data is added to an archive, we have other important design decisions to consider. I want to voice my agreement with several qualitative scholars who argue that while we should have ethical standards and design best practices in preserving and publishing some aspects of archival data, flexibility for the researcher is very necessary (Bishop, 2005; Jacobs et al., 2021). Recent qualitative data archives present an opportunity for exploratory and comparative research – something desperately needed for early career scholars, remote working scholars, and historical scholars. I support the efforts to control sensitive data, but there should also be space for more general access to less sensitive data. The risk around publishing data is on a spectrum and I am interested in continuing to explore what is possible through more public exchange of appropriate kinds of qualitative data.

In retrospect, I could have designed policies before I published the data on how involved with the distribution and explanation of the data I would be after I published it. I decided to participate very actively and answer all the questions that came my way because I saw this was an interesting opportunity to observe what happens to the data in its second life. I also saw my presence in and around the data as a necessary piece of context. I am always excited to collaborate with other social scientists, so I embraced this ongoing experiment as an opportunity to learn about unplanned collaboration.

Finally, if the co-production of the primary data occurs between the interviewer and the respondent, I would argue a much larger team is involved in producing the secondary archival data. Each team member (in our case, the interviewer, respondent, transcriber, markers, archive curator, archive editor, and librarians who serve as on-going stewards for the data) offers an important skill and knowledge that is a valuable part of the data preservation, and their needs should be considered and negotiated into the final secondary product with the privacy/wishes of the respondent serving as the priority consideration.

As we move forward with more collaborative social science programs, the issue of diversity within our conversations becomes increasingly important. Though we might be able to rely on multiple coders to capture varied interpretations of fact with a team, when our team offers similar perspectives on the field site and the data, we only capture some of the diversity necessary to understand the scope of the field. Some qualitative researchers already develop collaborative relationships with individuals deeply enmeshed in their field sites and ask for their opinions on the researcher’s analysis from the field (Duneier, 1999). However, we often draw our collaborators from our social networks, which may mean our overlapping similarities still cause us to miss important context within unfamiliar environments. For example, the debates and expectations around patterns of clear responses and direct communication emphasized in the US may not be an option or social norm in other environments. Reaching beyond our existing networks may help us develop more interesting conclusions than we can see from our current dominant perspectives. For example: styles of partially direct, partially indirect dialogue are very common in Mexico today in discussions of local gang/cartel violence, but they are also visible in the ways Eastern Europeans in the USSR described communication patterns, how members of organized crime units signal membership, and how many artists engage in public discussions of difficult politics without directly drawing the attention of those they critique (Gambetta, 2009). Information that may seem minor to an outsider may be deeply meaningful and relevant to someone with a deeper knowledge of the context. Maybe the best way to include them in our research process is to invite them to engage with more of our data and not just our analyses. There are many other places where the social norms of other cultures may help us improve our interpretations of data and sensemaking.

In summary: I am eager to see how other scholars design their studies with the intent to create public qualitative data sets. I am eager to see more literature reviews that blend the analysis of scholars with the analysis of respondents in existing interviews. I encourage and embrace decontextualized and triangulated interpretations of my interview data – especially because each student I met through my public data this past year taught me new ways to understand where my research could go next.

## Data availability statement

The datasets presented in this study can be found in online repositories. The names of the repository/repositories and accession number(s) can be found at: https://doi.org/10.34770/4324-yn77.

## Ethics statement

The studies involving humans were approved by Princeton University Institutional Review Board. The studies were conducted in accordance with the local legislation and institutional requirements. The participants provided their written informed consent to participate in this study.

## Author contributions

DE conducted the fieldwork and analyzed the data for the original publication, prepared the transcripts for publication, and published them through the Princeton Data Space. She is the main point of contact for individuals working with the data who have questions. JV was the PI on the project and coded the interview transcripts for the original publications drafted using this data. JV also hired different undergraduate research assistants to help anonymize the data before DE took a final read through the transcripts and published them. JV was consulted on a final copy of this article before DE submitted it for review.

## Footnotes

^1^More about Story Corps: https://storycorps.org/.

^2^More about the BBC Listening Project: https://www.bbc.co.uk/programmes/b01cqx3b.

^3^More about NPR: Story Corps https://www.npr.org/series/4516989/storycorps.

^4^Jason Del Rey, “Leaked Amazon Memo Warns the Company is Running Out of People to Hire,” Vox, June 17, 2022, https://www.vox.com/recode/23170900/leaked-amazon-memo-warehouses-hiring-shortage.

^5^I included my interview guide and consent form draft in the Appendix.

^6^Owens describes recruiting subjects for her study through public housing projects, which implies a threshold for how much this individual could earn annually before they were no longer eligible for this kind of housing. My subject was recruited through an ad placed on app for recipients of SNAP and/or WIC benefits, also implying this individual earned under a specific threshold set for those who qualify for state sponsored food subsidies.

^7^Princeton Research Data Service. “About DataSpace.” Online repository, 2022. https://researchdata.princeton.edu/research-lifecycle-guide/about-dataspace.
