# Supplementary material for: Publishing publicly available interview data: an empirical example of the experience of publishing interview data
Source: Front Sociol. 2024 Jun 5;9:1157514. doi: 10.3389/fsoc.2024.1157514 (PMC11188393; doi:10.3389/fsoc.2024.1157514)
Supplement: Supplementary file 1 [file Data_Sheet_1.pdf]

Publishing Publicly Available Interview Data:

An empirical example of the experience of publishing interview data

Diana Enriquez[1]*

[1] Department of Sociology, Princeton University, Princeton, New Jersey, USA

*** Correspondence:**
Diana Enriquez
de8@princeton.edu

Keywords: Open source, Qualitative methods, Interview data, Secondary data

Abstract

In September 2021 I made a collection of interview transcripts available for public use under a CreativeCommons license through the Princeton DataSpace. The interviews include 39 conversations I had with gig workers at AmazonFlex, Uber, and Lyft in 2019 as part of a study on automation efforts within these organizations. I made this decision because 1) I was required to contribute to a publicly available data set as a requirement of my funding and 2) I saw it as an opportunity to engage in the collaborative qualitative science experiments emerging in Science and Technology studies. This article documents my thought process and step-by-step design decisions for designing a study, gathering data, masking it, and publishing it in a public archive. Importantly, once I decided to publish these data, I determined that each choice about how the study would be designed and implemented had to be assessed for risk to the interviewee in a very deliberate way. It is not meant to be comprehensive and cover every possible condition a researcher may face while producing qualitative data. I aimed to be transparent both in my interview data and the process it took to gather and publish these data. I use this article to illustrate my thought process as I made each design decision for this study in hopes that it could be useful to a future researcher considering their own data publishing process.

APPENDIX

In the interest of transparency, I wanted to include some of the specific materials from my own public data project for reference. I am including my 1) interview guide and 2) consent form.

A. DISCUSSION GUIDE

I included part of the Discussion guide to show the types of questions I asked my interviewees, to demonstrate that my questions were focused on career and technology rather than more sensitive identifying questions. Once I posted the interviews online, I included my interview questions in the ReadMe published with the data to make it easier for someone coding the interviews to work with my data.

DISCUSSION GUIDE

[Consent form read by the interviewer to the interviewee before the interview began. The interviewee provided verbal consent before the interview began. A written document with the consent form information and contact information was provided by email before the interview.]

INTRO TO GIG WORK ROLE:

1. What is your job?

2. How did you find this job?

3. What other companies do you work for?

4. What do you do on those platforms?

5. How long have you worked for (this employer)?

6. What kind of introduction to the job or training did you receive for this job?

OTHER JOBS CONTEXT

7. How many jobs do you have?

8. How do you find these other jobs?

9. How do you structure your week between different jobs?

10. How many hours do you try to work per week?

11. How much do you get paid an hour?

12. Do you have a financial goal you try to hit for the day or week?

STRUCTURE OF ROLE

13. Who is your manager?

14. What do they do as their job?

15. Did you have to sign a contract to work for [COMPANY]?

16. Do you remember anything that contract said?

17. What skills do you need, or have you developed to do this job?

DATA and TECHNOLOGY

18. Is there any data that they record or track while you work?

19. Do you check in with someone on completed tasks?

20. What devices or tools do you use to do this job?

21. Have you noticed any new features or software in the app this year?

22. Has anything changed since you first started working for them?

23. How does this compare to other work that you've done?

WORK AND CAREER

24. Do you consider yourself an employee, self-employed, or running a small business? Why?

25. Do you expect to still be doing this job in a few years?

26. Are there other jobs you'd like to do in the future?

FUTURE OF TECH

27. How do you feel about self-driving cars?

28. How would you feel if [COMPANY] was going to start using self-driving cars in their fleet?

29. Does it seem like that is something [COMPANY] might do?

B. CONSENT FORM

The consent form explains how I asked my interviewees for consent to publish the interview transcript as part of a public data set after the project was completed. I sent this form to their email addresses and read it to them before we began our interview. I decided to ask for consent twice to make sure they understood the public nature of my data set and how I would handle their privacy:

What do I need to do? Please answer our questions as honestly as you can. If you do not feel comfortable answering a question, just ask us to skip it. If you want to stop at any time, just tell us "Stop."

Will this be risky or harmful to me? We do not think so. We will make sure that your name is hidden, and we will anonymize you and anyone else you name in this study. And we will not tell your employer that you spoke to us. That information is confidential and protected. Your participation will not affect your employment eligibility or your immigration status.

Why should I participate? Our study will help everyone to know more about "the gig economy" and the experiences of workers on the ground. It will also provide valuable information for others studying "gig work" to understand how this is impacting our economy and our society.

Confidentiality: All records from this study will be kept confidential. Your responses will be private. We will not include any information that will identify you in any report we might publish.

About this data: We will release the transcripts from our conversations to researchers to use. We think this information is valuable for others to know what your experiences are, and to know that our findings are correct. We will make every effort to be sure that no one can identify you in the data we release.

Compensation: We will pay $15 in a gift card to you for your time.

Questions: If you have any questions about your rights, your information, or our study you may contact (PI) at (office phone number), or (email address). If you experience problems with the study team, please contact Princeton's Institutional Review Board at (Phone number), or (email).
